# ﻿A new species and two new records of the moss-feeding lace bug genus *Acalypta* (Hemiptera, Heteroptera, Tingidae) from Hokkaido, northern Japan, with an illustrated key to the Japanese species of the genus

**DOI:** 10.3897/zookeys.1229.142344

**Published:** 2025-02-26

**Authors:** Jun Souma

**Affiliations:** 1 Shirakami Research Center for Environmental Sciences, Faculty of Agriculture and Life Science, Hirosaki University, Aomori, Japan Hirosaki University Aomori Japan

**Keywords:** Checklist, East Asia, host plant, phytophagous insect, lace bug, taxonomy

## Abstract

In this study, three species of the moss-feeding lace bug genus *Acalypta* Westwood, 1840 (Hemiptera, Heteroptera, Tingidae, Tinginae, Acalyptaini) are reported mainly in Hokkaido, northern Japan. The first is *Acalyptaalutacea***sp. nov.** that inhabits mosses growing on the floors of deciduous broad-leaved forests. The second is *Acalyptacarinata* (Panzer, 1806) that mainly inhabits mosses growing on marshlands and is recorded from Japan for the first time. The third is *Acalyptasauteri* Drake, 1942 that is widely distributed in three of the four main islands of Japan (Honshu, Shikoku, and Kyushu) and their surrounding islands, but is recorded from the remaining main island (Hokkaido) for the first time. The discovery of *A.sauteri* in Hokkaido also represents the northernmost record of this species. The following ten species of *Acalypta* are recognized in Japan: *A.alutacea***sp. nov.**, *A.carinata*, *A.cooleyi* Drake, 1917, *A.gracilis* (Fieber, 1844), *A.hirashimai* Takeya, 1962, *A.marginata* (Wolff, 1804), *A.miyamotoi* Takeya, 1962, *A.pallidicoronata* Souma, 2019, *A.sauteri*, and *A.tsurugisana* Tomokuni, 1972. An illustrated key for the identification of the ten species of this genus from Japan is also provided.

## ﻿Introduction

Lace bugs (Hemiptera, Heteroptera, Tingidae) are phytophagous true bugs that are highly host specific and generally feed on the abaxial sides of angiosperm leaves (Schuh and Weirauch 2020). The genus *Acalypta* Westwood, 1840 (Tinginae, Acalyptaini), a unique genus of lace bugs associated with mosses, comprises 46 species in the northern hemisphere (cf. Drake and Lattin 1963; Drake and Ruhoff 1965; Tomokuni 1983; Schuh and Weirauch 2020; Souma 2019, 2023a). In Japan, the following eight species have been recorded from the four main islands belonging to the Palaearctic Region (Hokkaido, Honshu, Shikoku, and Kyushu) and its surrounding islands: *A.cooleyi* Drake, 1917; *A.gracilis* (Fieber, 1844); *A.hirashimai* Takeya, 1962; *A.marginata* (Wolff, 1804); *A.miyamotoi* Takeya, 1962; *A.pallidicoronata* Souma, 2019; *A.sauteri* Drake, 1942; and *A.tsurugisana* Tomokuni, 1972 (Yamada and Ishikawa 2016; Souma 2019, 2020, 2021, 2023a). In the past six years, many distributional records of *Acalypta* species from Japan have been published (Maehara 2019; Obae 2019; Okochi 2019, 2023; Yamaguchi and Nakamura 2019; Yoshitomi and Hayashi 2019; Kawase 2020; Konno 2020, 2023; Yamamoto 2021a, 2021b, 2022a, 2022b, 2024a, 2024b; Nakamura 2022; Takai 2022; Souma 2019, 2020, 2021, 2022, 2023a, 2023b; Ban et al. 2024) owing to increased public awareness, but most reports include only *A.cooleyi*, *A.miyamotoi*, *A.pallidicoronata*, *A.sauteri*, and/or *A.tsurugisana* Tomokuni, 1972, which are distributed in one of the three main islands of Japan (Honshu, Shikoku, and Kyushu) and/or their surrounding islands (Yamada and Tomokuni 2012; Yamada and Ishikawa 2016; Souma 2023a). On the remaining main island located in northern Japan (Hokkaido), only three species, namely, *A.gracilis*, *A.hirashimai*, and *A.marginata*, are known at present (Yamada and Ishikawa 2016; Souma 2020, 2021) and are not distributed in the other three main islands. However, most previous studies on *Acalypta* species from Hokkaido have reported only *A.hirashimai*, and distributional records for *A.gracilis* and *A.marginata* are sparse (cf. Takeya 1962; Tomokuni 1972, 1983, 1987a, 1987b; Iijima 2006; Yamamoto 2006; Souma 2020, 2021, 2023a). In contrast to studies on lace bugs in the other three main islands of Japan, only a few studies have been published on *Acalypta* species from Hokkaido based on recently collected specimens (cf. Souma 2020, 2023a). Therefore, *Acalypta* species from Hokkaido have been poorly studied despite increased public awareness, and further field surveys may reveal unexpected species.

An unidentified species of *Acalypta* was collected from Hokkaido in 2015 and 2017 (Nakatani 2016, 2018). During a recent field survey in Hokkaido, the author collected additional individuals of this indeterminate species from mosses growing on the forest floor of deciduous broad-leaved forests in 2024, and another indeterminate species from those growing on marshlands in 2023. Moreover, the author’s colleague, Ryoichi Sato, collected *A.sauteri* from Hokkaido in 2024, which has not been previously recorded on this island. After careful examination of the morphological characteristics, the author concluded that one indeterminate species recorded by Nakatani (2016, 2018) represents an undescribed species, and another indeterminate species corresponds to *A.carinata* (Panzer, 1806), which is widely distributed in the Palaearctic Region (Péricart and Golub 1996; Aukema et al. 2013). While this study was almost completed, the author’s colleague, Ryotaro Wakimura, collected *A.carinata* from mosses growing on the forest floor of deciduous broad-leaved forests on Rishiri Island, near Hokkaido.

In this study, the author describes a new species, *Acalyptaalutacea* sp. nov., and newly records *A.carinata* and *A.sauteri* from Japan and Hokkaido, respectively. Additionally, an illustrated key for the identification of all ten species of *Acalypta* occurring in Japan has been provided. Furthermore, a checklist and photographs of living individuals of the ten Japanese species of *Acalypta* are also presented.

## ﻿Materials and methods

Morphological characteristics of the specimens were observed, drawn, and measured using a stereoscopic microscope (SZX16; Olympus, Tokyo, Japan) equipped with an ocular grid. To examine the male genitalia, the pygophore was removed from the body after softening the specimens in hot water. The removed pygophore was immersed in hot 15% potassium hydroxide (KOH) solution for 5 min. The paramere was immersed in 99% ethanol and removed from the genital capsule for further observation. Male genitalia were preserved in small polyethylene vials containing a 50% aqueous glycerin solution. Male genitalia were viewed after the angles were fixed with gel (Museum Gel Clear; Ready America, California, USA) and placed on a microscope slide. A polyethylene vial was mounted on the pin containing the specimens. The specimens were photographed using a digital camera (EOS 90D; Canon, Tokyo, Japan) equipped with a zoom lens (18–35 mm F1.8 DC HSM; SIGMA, Kanagawa, Japan) and a digital microscope (Dino-Lite Premier M; Opto Science, Tokyo, Japan). Photographs of the living individuals were captured using a compact digital camera (Tough TG-6; Olympus, Tokyo, Japan). The image stacks of the specimens were processed using a Zerene Stacker (Zerene Systems, Washington, USA). All illustrations and photographs were processed using Adobe Photoshop 2024 ver.25.11. Morphological terms were assigned according to previous monographs (Takeya 1962; Drake and Davis 1960; Drake and Ruhoff 1965; Schuh and Weirauch 2020).

The specimens examined in this study were deposited at the Shirakami Research Center for Environmental Sciences, Faculty of Agriculture and Life Science, Hirosaki University, Aomori, Japan (**SIHU**) and Laboratory of Entomology, Faculty of Agriculture, Tokyo University of Agriculture, Kanagawa, Japan (**TUA**).

Species distribution records were mapped using SimpleMappr (Shorthouse 2010). Geographic coordinates were obtained from Google Maps (https://www.google.co.jp/maps). The map was edited using the Adobe Photoshop software. The scientific names of the host plants were assigned according to Yonekura and Kajita (2003–2024).

## ﻿Systematics

### ﻿Description of new species

#### ﻿Genus Acalypta

Taxon classificationAnimaliaHemipteraTingidae

Westwood, 1840

13EE1A69-8E1F-5BA9-AEB3-885895437871

Acalypta Westwood, 1840: 121. Type species by original designation: Tingiscarinata Panzer, 1806.

##### Note.

For synonyms and detailed descriptions of the genus see Drake and Lattin (1963), Péricart (1978, 1983), and Péricart and Golub (1996).

##### Remarks.

The genus *Acalypta*, which is distributed only in the northern hemisphere, previously comprised 46 species (cf. Souma 2019, 2023a), but in this study, the author describes a new species, *A.alutacea* sp. nov., increasing the number of species to 47. Among them, the following eight species have been known from Japan: *A.cooleyi*; *A.gracilis*; *A.hirashimai*; *A.marginata*; *A.miyamotoi*; *A.pallidicoronata*; *A.sauteri*; and *A.tsurugisana* (Yamada and Ishikawa 2016; Souma 2019, 2020, 2021, 2023a). However, with the description of *A.alutacea* sp. nov. and the first record of *A.carinata* from Japan, a total of ten *Acalypta* species are currently recognized.

#### ﻿ Acalypta alutacea sp. nov.

Taxon classificationAnimaliaHemipteraTingidae

8A245ADF-197F-5E39-9644-5568C1814AF1

https://zoobank.org/5C85303C-85C5-403F-B4F3-E3602DA61AAC

Figs 1A, B , 3A–D , 5A , 7A–D , 9A , 11A , 13A, B , 14A, B , 15A Japanese name: Kushiromaru-gunbai

Acalypta sp.: Nakatani (2016: 50) (distribution); Nakatani (2018: 48) (distribution).

##### Material examined.

***Holotype***, Japan • brachypterous ♂; Hokkaido, Kushiro-gun, Kushiro-cho, Beppo; 27 Jun. 2024; J. Souma leg.; SIHU. ***Paratypes***, Japan • 1 brachypterous ♂ 4 brachypterous ♀♀; same data as for holotype; SIHU • 1 brachypterous ♂ 1 brachypterous ♀; “春採湖北岸” [= Hokkaido, Kushiro-shi, Shunkodai, North shore of Harutori Lake]; 8 Jul. 2015; M. Nakatani leg.; SIHU. Two specimens collected in 2015 were recorded as “*Acalypta* sp.” in a previous study (Nakatani 2016).

##### Diagnosis.

**Figure 1.**
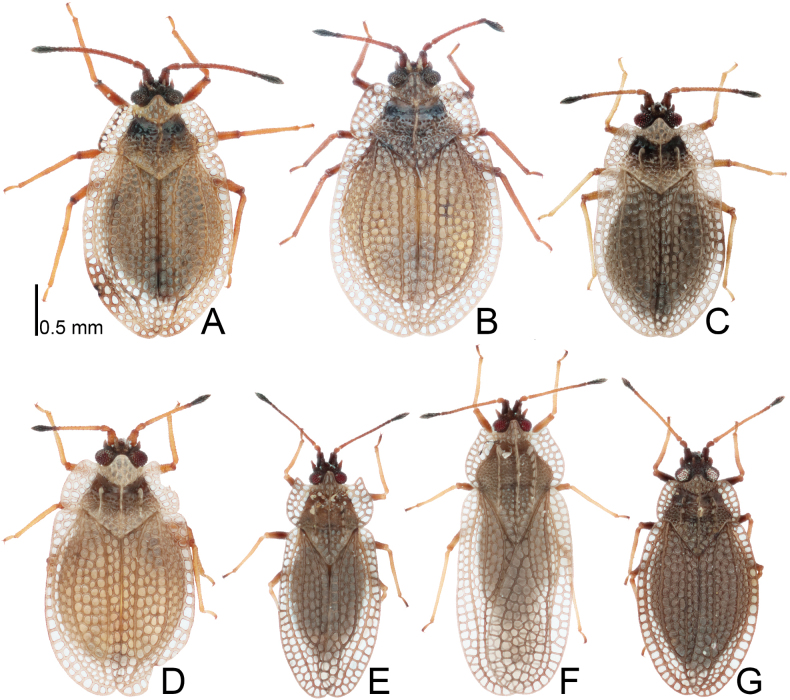
Dorsal habitus of four *Acalypta* species from Japan. **A***A.alutacea* sp. nov., brachypterous male **B***A.alutacea* sp. nov., female **C***A.carinata*, brachypterous male **D***A.carinata*, brachypterous female **E***A.cooleyi*, brachypterous male **F***A.cooleyi*, macropterous male **G***A.gracilis*, brachypterous male.

*Acalyptaalutacea* sp. nov. is recognized among the other species of *Acalypta* based on a combination of the following characteristics: brachypterous morph only known; hemelytron without dark spots (Fig. 1A, B); antenniferous tubercle obtuse, curved inward (Figs 3A–D, 13A, B); basal part of antennal segment III not thickened; rostrum reaching anterior part of abdominal sternite II (Fig. 5A); pronotum 3/4 times as long as maximum width across paranota; pronotal disc as long as calli; punctures on pronotal disc smaller than areolae of posterior process; posterior part of hood usually triangular; lateral carina of pronotum absent or reduced, shorter than hood, without or with a single minute areola; calli smooth; paranotum as wide as hood, not narrowed posteriorly, with 3–4 rows of areolae throughout its length; anterolateral angle of paranotum rounded, not or weakly protruding anteriorly, not reaching mid-level of compound eye; posterolateral angle of paranotum protruding posteriorly; posterior process strongly protruding posteriorly, as long as hood; costal area of hemelytron with 2–3 rows of areolae in basal part, a single row in middle part, and 1–2 rows in apical part (Fig. 7A–D); discoidal area expanded beyond apical fourth of hemelytron, wider than subcostal area; Cu (cubitus) vein distinct throughout its length; abdomen pale brown in female (Fig. 11A); and pygophore roundly inflated (Fig. 14A).

##### Description.

***Brachypterous male*.** Frontal spine of head, pronotum except for calli, hemelytron, and sternal laminae pale brown; antenniferous tubercle, antennal segments I to III, ventral surface of thorax except for sternal laminae, and legs brown; most parts of head, antennal segment IV, calli, and abdomen dark brown; paranotum and hemelytron sometimes irregularly dark in very small sections, without dark spots; areolae of pronotum and hemelytron transparent; compound eyes dark red; pubescence on body yellowish (Figs 1A, 3A, B, 5A, 7A, B, 9A).

Body ovate; pubescence on most parts of body distinctly shorter than radius of compound eye (Figs 1A, 3A, B, 5A, 9A). Head covered with minute pubescence; pair of frontal spines separated at apices, reaching apex of clypeus; antenniferous tubercle obtuse, curved inward, shorter than frontal spine; vertex and clypeus smooth. Compound eye round in dorsal view. Antenna densely covered with minute pubescence in segments I to III and long pubescence in segment IV; pubescence on segment IV longer than pubescence on other parts of body; segment I cylindrical, shorter than segment IV; segment II conical, shortest amongst antennal segments; segment III linear, longest amongst antennal segments, not thickened in basal part; segment IV fusiform. Bucculae closed at anterior ends, with 3 rows of areolae at highest part. Rostrum reaching anterior part of abdominal sternite II.

Pronotum (Figs 3A, B, 13A, B) glabrous, 3/4 times as long as maximum width across paranota. Pronotal disc coarsely punctate, as long as calli; punctures smaller than areolae of posterior process. Hood roof-shaped, shorter than median carina of pronotum, wider than vertex at widest part, not covering compound eye, with 5 rows of areolae at highest part; posterior part usually triangular. Median carina straight, extending to apex of posterior process, with a single row of areolae throughout its length. Lateral carina absent or reduced, shorter than hood, without or with a single minute areola. Calli smooth. Paranotum horizontally expanding outward, as wide as hood, not narrowed posteriorly, with 3–4 rows of areolae throughout its length; anterolateral angle rounded, not or weakly protruding anteriorly, not reaching mid-level of compound eye; posterolateral angle protruding posteriorly. Posterior process triangular, obtuse at apex, strongly protruding posteriorly, as long as hood.

Hemelytron (Fig. 7A, B) glabrous, extending beyond apex of abdomen; apex close to the other at rest; costal area with 2–3 rows of areolae in basal part, a single row in middle part, and 1–2 rows in apical part; subcostal area with 4 rows of areolae at widest part; discoidal area expanded beyond apical fourth of hemelytron, wider than subcostal area, with 4–5 rows of areolae at widest part; sutural area with 3 rows of areolae at widest part; hypocostal lamina with a single row of areolae throughout its length; Sc (subcosta), Hc (hypocosta), R+M (fused radius and media) and Cu (cubitus) veins distinct throughout their respective length.

Thoracic pleura (Fig. 5A) smooth in anterior part, coarsely punctate in posterior part. Ostiolar peritreme reduced. Mesosternum narrower than metasternum. Sternal laminae lower than buccula, open in both anterior and posterior ends; metasternal lamina as high as mesosternal lamina. Legs (Fig. 1A) smooth, covered with minute pubescence; femora thickest at middle.

Abdomen ovate in dorsal and ventral views, glabrous except for terminalia, smooth. Pygophore (Figs 9A, 14A) roundly inflated, semicircular in ventral view, covered with minute pubescence; anterior margin concave in middle part. Paramere (Fig. 14B) slender, expanded in middle part, gently curved inward, covered with minute pubescence in middle part of outer and inner margins.

Measurements (*n* = 3). Body length with hemelytra 2.30–2.60 mm; maximum width across hemelytra 1.30–1.50 mm; length of antennal segments I to IV 0.15 mm, 0.10 mm, 0.70–0.80 mm, and 0.20–0.25 mm, respectively; pronotal length 0.70–0.80 mm; pronotal width across paranota 1.10–1.20 mm; hemelytral length 1.65–1.85 mm; maximum width of hemelytron 0.70–0.80 mm.

***Brachypterous female*.** General habitus very similar to that of male (Figs 1B, 3C, D, 7C, D, 11A) except for the following characters: abdomen pale brown; body usually longer and wider than in male; antennal segment III shorter than in male; and apical part of abdomen pentagonal in ventral view.

Measurements (*n* = 5). Body length with hemelytra 2.60–2.80 mm; maximum width across hemelytra 1.65–1.80 mm; length of antennal segments I to IV 0.15 mm, 0.10 mm, 0.60 mm, and 0.20 mm, respectively; pronotal length 0.80–0.90 mm; pronotal width across paranota 1.20–1.30 mm; hemelytral length 1.90–2.00 mm; maximum width of hemelytron 0.85–0.95 mm.

##### Remarks.

Among all the species of *Acalypta*, *A.alutacea* sp. nov. strongly resembles *A.carinata* and *A.platycheila* (Fieber, 1844) in terms of its general habitus. However, based on a comparison between the type materials of the new species and the non-types and descriptions (Péricart 1978, 1983) of *A.carinata* and *A.platycheila*, five main characteristics were recognized to easily differentiate *A.alutacea* sp. nov. from *A.carinata* and *A.platycheila* (Figs 1A–D, 3A–G, 7A–G, 13A–D): posterior part of pronotal hood usually triangular (usually semicircular in *A.platycheila*); lateral carinae absent or reduced, shorter than hood, without or with a single minute areola (developed, as long as hood, with a single row of areolae in *A.carinata* and *A.platycheila*); calli smooth (rough in *A.platycheila*); anterolateral angle of paranotum not or weakly protruding anteriorly, not reaching mid-level of compound eye (strongly protruding anteriorly, reaching mid-level of compound eye in *A.carinata* and *A.platycheila*); and costal area of hemelytron with 2–3 rows of areolae in basal part, a single row in middle part, and 1–2 rows in apical part (with usually a single row throughout its length and sometimes 2 rows in basal part in *A.platycheila*). Morphological differences between the new species and the eight other Japanese species besides *A.carinata* are presented in the identification key below.

##### Distribution.

Japan (Hokkaido) (Fig. 17) (Nakatani 2016, 2018).

##### Etymology.

The species epithet is the Latin adjective “alutaceus”, referring to the pale brown body color.

##### Host plant.

Six individuals of *Acalyptaalutacea* sp. nov. were collected from indeterminate mosses growing on the forest floor; at least one of these mosses could be a host plant for the new species.

##### Bionomics.

*Acalyptaalutacea* sp. nov. inhabits the forest floor of deciduous broad-leaved forests in Hokkaido, which has a cool temperate climate.

Adults were collected in June and July (Nakatani 2016, 2018) but nymphs were not collected.

### ﻿New records of *Acalypta* species from northern Japan

#### ﻿ Acalypta carinata

Taxon classificationAnimaliaHemipteraTingidae

(Panzer, 1806)

E3A239E1-9528-595F-AC3C-9484B4E62C9B

Figs 1C, D , 3E–G , 5B , 7E–G , 9B , 11B , 13C, D , 15B–D Japanese name: Yachimaru-gunbai

Tingis carinata Panzer, 1806: 631. Syntype(s): sex unknown; type locality: Germany • “Mannheimii” [= Mannheim]; depository: unknown.
Acalypta carinata : Westwood (1840: 121) (new combination).

##### Note.

For synonyms and detailed descriptions of the species see Drake and Ruhoff (1965), Péricart (1978, 1983), and Péricart and Golub (1996).

##### Material examined.

***Non-types***, Japan • 1 brachypterous ♀; Hokkaido, Soya-gun, Sarufutsu-mura, Sarufutsu; 15 Aug. 2023; J. Souma leg.; SIHU • 3 brachypterous ♂♂ 1 brachypterous ♀ 7 fifth instar nymphs; same collection data as for preceding; 29 Sep. 2023; adults have emerged until 26 Oct. 2023 by fed on mosses growing on marshlands in captivity; SIHU • 1 brachypterous ♂ 1 brachypterous ♀ 2 fifth instar nymphs; Rishiri Island, Rishirifuji-cho, Oshidomari; 28 Sep. 2024; R. Wakimura leg.; adults have emerged until 15 Nov. 2024 by fed on mosses growing on forest floor of deciduous broad-leaved forests in captivity; SIHU • 1 brachypterous ♀ 1 fifth instar nymph; Rishiri Island, Rishiri-cho, Kutsugata; 29 Sep. 2024; R. Wakimura leg.; adult has emerged until 30 Nov. 2024 by fed on mosses growing on forest floor of deciduous broad-leaved forests in captivity; SIHU.

##### Remarks.

The 18 specimens recorded above (Fig. 1C, D) matched well with the descriptions and illustrations of *Acalyptacarinata* (Péricart 1978, 1983) in terms of their morphological characteristics, particularly the structures of the pronotum and hemelytron (Figs 3E–G, 7E–G, and Fig. 13C, D). Therefore, the specimens examined were identified as *A.carinata*.

*Acalyptacarinata* is most similar to *A.platycheila* in general appearance, but the former can be distinguished from the latter based on the following characters (Figs 3E–G, 7E–G, 13C, D) (cf. Péricart 1978, 1983): posterior part of pronotal hood usually triangular (usually semicircular in *A.platycheila*); lateral carinae slightly close to each other toward anterior ends (parallel or slightly separated in *A.platycheila*); calli smooth (rough in *A.platycheila*); and costal area of hemelytron with usually 2 rows of areolae throughout its length, sometimes 3 rows in basal part, and sometimes a single row in middle part (with usually a single row throughout its length and sometimes 2 rows in basal part in *A.platycheila*). Morphological differences between *A.carinata* and the nine other Japanese species are presented in the identification key below.

The teratological form of the pronotum and hemelytron was confirmed in *A.carinata* from Japan, and one examined specimen possessed malformations of the right paranotum and hemelytron (Figs 1D, 3G, 7G), as reported in many lace bugs (Štusák and Stehlík 1980, 1982).

##### Distribution.

Austria, Belgium, Byelorussia, Croatia, Czech Republic, Denmark, Estonia, Finland, France, Great Britain, Germany, Hungary, Ireland, Italy, Japan (Hokkaido, Rishiri Island), Latvia, Luxembourg, Moldovia, Mongolia, Netherlands, Norway, Poland, Romania, Russia, Slovakia, Slovenia, Spain, Sweden, Switzerland (Fig. 17) (Péricart and Golub 1996; Aukema et al. 2013; Aukema 2025). *Acalyptacarinata* is newly recorded from Japan.

##### Host plant.

The Japanese population of *Acalyptacarinata* feeds on indeterminate mosses growing in marshlands or the forest floor of deciduous broad-leaved forests in captivity. In other distribution areas, *Abietinellaabietina* (Hedw.) M.Fleisch. (Thuidiaceae), and indeterminate mosses have been recorded as host plants for this species (cf. Drake and Ruhoff 1965; Péricart 1983).

##### Bionomics.

*Acalyptacarinata* is found only in marshlands in Hokkaido, Japan and is dominant among *Acalypta* species in humid environments, such as marshlands and riparian forests in Hungary (Rédei et al. 2004), suggesting that it mainly inhabits humid environments regardless of the region. However, this species inhabits the forest floor of deciduous broad-leaved forests exceptionally on Rishiri Island, near Hokkaido, where no other *Acalypta* species has been recorded.

In Japan, adults and nymphs were observed in August and September, respectively. In Europe, adults were collected between April and October, and nymphs were confirmed in almost all seasons; the overwintering stage comprised nymphs (cf. Péricart 1983; Rédei et al. 2004).

#### ﻿ Acalypta sauteri

Taxon classificationAnimaliaHemipteraTingidae

Drake, 1942

78A92F96-FB65-57BC-83B4-504429BB3B11

**Figure 2.**
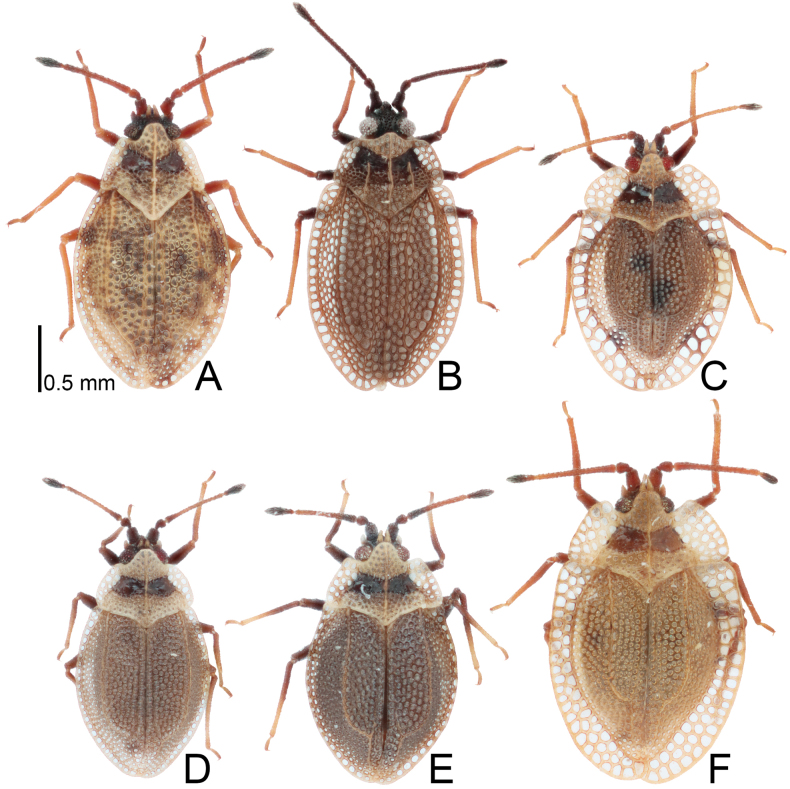
Dorsal habitus of six *Acalypta* species from Japan. **A***A.hirashimai*, brachypterous male **B***A.marginata*, brachypterous male **C***A.miyamotoi*, brachypterous male **D***A.pallidicoronata*, brachypterous male **E***A.sauteri*, brachypterous male **F***A.tsurugisana*, brachypterous male.

Figs 2E , 4E , 6C , 8E , 10C , 12C , 16C, D Japanese name: Maru-gunbai

Acalypta sauteri Drake, 1942: 14. Holotype: brachypterous ♂; type locality: Japan • Oayama [= Honshu, Kanagawa-ken, Mt. Oyama] (cf. Takeya 1951; Miyatake 1958; Hiura 1960; Drake and Lattin 1963; Tomokuni 1983); depository: United States National Museum of Natural History, Washington, D.C., USA.

##### References.

Takeya (1951: 6) (checklist: eastern Asia); Miyatake (1958: 15) (distribution); Hiura (1960: 70) (distribution); Takeya (1962: 68) (distribution); Drake and Lattin (1963: 336) (distribution); Drake and Ruhoff (1965: 54) (catalogue); Miyamoto (1965: 90) (monograph); Tomokuni (1972: 88) (distribution); Baba (1982: 8) (distribution); Tomokuni (1983: 146) (biology); Tomokuni (1985: 156) (distribution); Miyamoto and Yasunaga (1989: 166) (checklist: Japan); Takahashi (1990: 3) (distribution); Péricart and Golub (1996: 10) (checklist: Palaearctic); Miyamoto (2008: 157) (monograph); Yamada and Tomokuni (2012: 187) (monograph); Yano et al. (2013: 24) (distribution); Maehara (2014: 57) (distribution); Nozaki et al. (2016: 75) (distribution); Yamada and Ishikawa (2016: 429) (checklist: Japan); Hayashi and Kadowaki (2018: 309) (checklist: Oki Islands); Ito and Sasaki (2018: 17) (distribution); Obae (2019: 18) (distribution); Okochi (2019: 1) (distribution); Yamaguchi and Nakamura (2019: 61) (distribution); Yoshitomi and Hayashi (2019: 84) (distribution); Kawase (2020: 6) (distribution); Konno (2020: 18) (distribution); Yamamoto (2021a: 4) (distribution); Takai (2022: 61) (distribution); Konno (2023: 18) (distribution); Okochi (2023: 5) (distribution); Yamamoto (2022a: 2) (distribution); Yamamoto (2022b: 10) (distribution); Ban et al. (2024: 115) (distribution); Yamamoto (2024a: 4) (distribution); Yamamoto (2024b: 9) (distribution).

##### Material examined.

***Non-types***, Japan • 1 brachypterous ♂; Hokkaido, Yakumo-cho, Namarikawa; 29 Jun. 2024; R. Sato leg.; SIHU • 2 brachypterous ♀♀; same collection data as for preceding; 6 Oct. 2024; SIHU • 1 brachypterous ♂ 1 brachypterous ♀; Awa Island, Uramura; 20 Sep. 2015; G. Mashima leg.; TUA.

**Figure 3.**
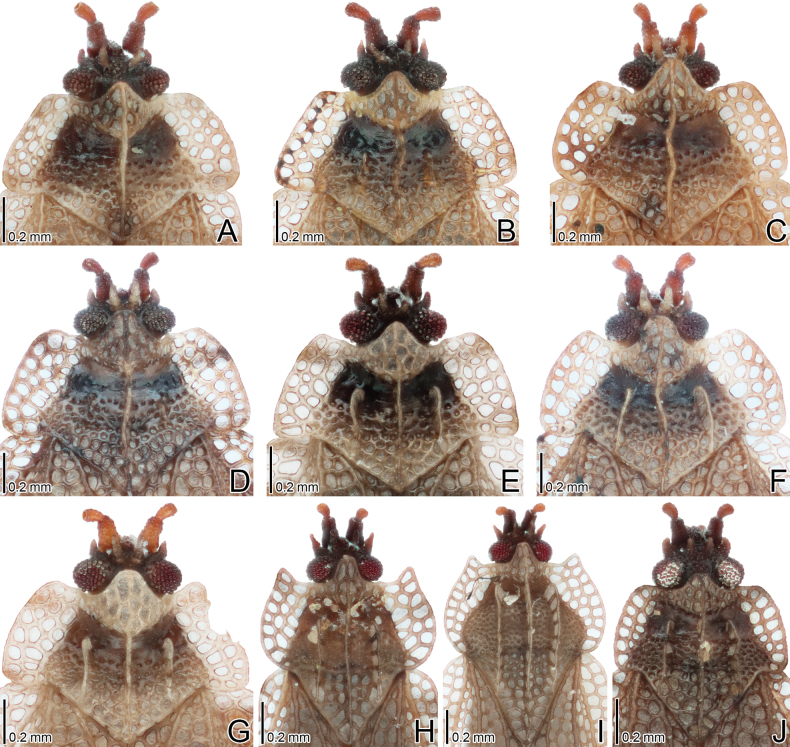
Pronota of four *Acalypta* species from Japan. **A, B***A.alutacea* sp. nov., brachypterous males **C, D***A.alutacea* sp. nov., brachypterous females **E***A.carinata*, brachypterous male **F, G***A.carinata*, brachypterous females **H***A.cooleyi*, brachypterous male **I***A.cooleyi*, macropterous male **J***A.gracilis*, brachypterous male.

##### Remarks.

Although *Acalyptasauteri* exhibits geographic variations (Tomokuni 1972), the morphological characteristics of the above five specimens were consistent with photographs of the holotype (United States National Museum of Natural History 2024) and the original description (Drake 1942) of the species. Therefore, we identified the studied specimens from Hokkaido and Awa Island as *A.sauteri*. Morphological differences between *A.sauteri* and nine other Japanese species are provided in the identification key below.

##### Distribution.

Japan (Hokkaido, Honshu, Awa Island, Sado Island, Awaji Island, Oki Islands (Dogo Island), Ikuchi Island, Shikoku, Shodo Island, Omi Island, Kyushu, Amakusa Islands (Shimoshima Island)) (Fig. 17) (Tomokuni 1983, 1985; Nozaki et al. 2016; Yamada and Tomokuni 2012; Yamada and Ishikawa 2016; Aukema 2025). *Acalyptasauteri* is newly recorded from Hokkaido and Awa Island. The discovery of *A.sauteri* from Hokkaido also represents the northernmost record of this species.

##### Host plant.

*Acalyptasauteri* were collected from 23 moss species belonging to 14 families: *Atrichumrhystophyllum* (Müll.Hal.) Paris (Polytrichaceae), *Brachytheciumbuchananii* (Hook.) A.Jaeger (Brachytheciaceae), *B.plumosum* (Hedw.) Bruch et Schimp, *B.populeum* (Hedw.) Bruch et Schimp., *B.rivulare* Bruch et Schimp., *Brotherellahenonii* (Duby) M.Fleisch. (Pylaisiadelphaceae), *Bryhniatrichomitria* Dixon et Thér. (Brachytheciaceae), *Codriophorusanomodontoides* (Cardot) Bednarek-Ochyra et Ochyra (Grimmiaceae), *Cratoneuronfilicinum* (Hedw.) Spruce (Amblystegiaceae), *Entodonsullivantii* (Müll.Hal.) Lindb. (Entodontaceae), *Haplocladiummicrophyllum* (Hedw.) Broth. (Leskeaceae), *Hypnumcupressiforme* Hedw. (Hypnaceae), *H.oldhamii* (Mitt.) A.Jaeger et Sauerb., *H.plumaeforme* Wilson, *H.tristoviride* (Broth.) Paris, *Kindbergiaarbuscula* (Broth.) Ochyra (Brachytheciaceae), *Leptodictyumriparium* (Hedw.) Warnst. (Amblystegiaceae), *Philonotisthwaitesii* Mitt. (Bartramiaceae), *Plagiomniumacutum* (Lindb.) T.J.Kop. (Mniaceae), *Plagiotheciumeuryphyllum* (Cardot et Thér.) Z.Iwats. (Symphyodontaceae), *Rosulabryumbillardieri* (Schwägr.) J.R.Spruce (Bryaceae), *Trachycystismicrophylla* (Dozy et Molk.) Lindb. (Mniaceae), and *Thuidiumkanedae* Sakurai (Thuidiaceae) (Tomokuni 1983, 1985; Yamada and Tomokuni 2012; Yamamoto 2022a; Ban et al. 2024).

##### Bionomics.

Adults were observed in almost all seasons, and nymphs were collected in January and May, and from August to October (Drake 1942; Takeya 1951, 1962; Miyatake 1958; Hiura 1960; Drake and Lattin 1963; Tomokuni 1972, 1983, 1985; Baba 1982; Takahashi 1990; Yamada and Tomokuni 2012; Yano et al. 2013; Maehara 2014; Nozaki et al. 2016; Ito and Sasaki 2018; Yamaguchi and Nakamura 2019; Obae 2019; Okochi 2019, 2023; Yoshitomi and Hayashi 2019; Kawase 2020; Konno 2020, 2023; Yamamoto 2021a, 2022a, 2022b, 2024a, 2024b; Takai 2022; Ban et al. 2024), suggesting that the overwintering stage is characterized by adults and nymphs.

### ﻿Checklist of *Acalypta* species occurring in Japan

***Acalyptaalutacea* sp. nov.**

Figs 1A, B, 3A–D, 5A, 7A–D, 9A, 11A, 13A, B, 14A, B, 15A

Japanese name: Kushiromaru-gunbai

**Distribution.** Japan (Hokkaido).

***Acalyptacarinata* (Panzer, 1806)**

Figs 1C, D, 3E–G, 5B, 7E–G, 9B, 11B, 13C, D, 15B–D

Japanese name: Yachimaru-gunbai

**Distribution.** Austria, Belgium, Byelorussia, Croatia, Czech Republic, Denmark, Estonia, Finland, France, Great Britain, Germany, Hungary, Ireland, Italy, Japan (Hokkaido, Rishiri Island), Latvia, Luxembourg, Moldovia, Mongolia, Netherlands, Norway, Poland, Romania, Russia, Slovakia, Slovenia, Spain, Sweden, Switzerland (Péricart and Golub 1996; Aukema et al. 2013; Aukema 2025).

***Acalyptacooleyi* Drake, 1917**

Figs 1E, F, 3H, I, 5C, 7H, I, 9C, 11C, 15E, F

Japanese name: Maeharamaru-gunbai

**Distribution.** China, Kazakhstan, Mongolia, Japan (Honshu), Russia, Tajikistan, USA (Péricart and Golub 1996; Aukema et al. 2013; Souma 2019; Aukema 2025).

***Acalyptagracilis* (Fieber, 1844)**

Figs 1G, 3J, 5D, 7J, 9D, 11D, 15G

Japanese name: Muromaru-gunbai

**Distribution.** Armenia, Austria, Azerbaijan, Belgium, Bulgaria, Byelorussia, Czech Republic, Denmark, Estonia, Finland, France, Georgia, Germany, Greece, Hungary, Italy, Japan (Hokkaido, Kunashiri Island), Latvia, Lithuania, Luxemburg, Macedonia, Moldavia, Mongolia, Netherlands, Norway, Poland, Romania, Russia, Serbia, Slovakia, Spain, Sweden, Switzerland, Tadzhikistan, Turkey, Turkmenistan, Ukraine (Kerzhner 1978; Péricart and Golub 1996; Aukema et al. 2013; Souma 2020; Aukema 2025).

***Acalyptahirashimai* Takeya, 1962**

**Figure 4.**
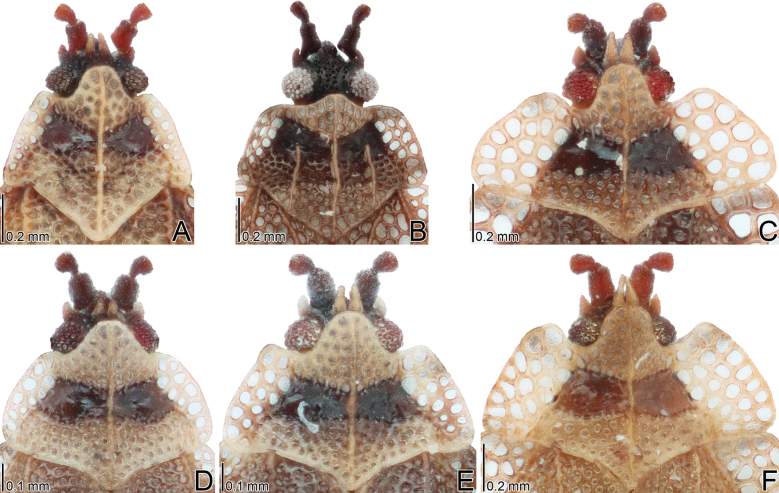
Pronota of six *Acalypta* species from Japan. **A***A.hirashimai*, brachypterous male **B***A.marginata*, brachypterous male **C***A.miyamotoi*, brachypterous male **D***A.pallidicoronata*, brachypterous male **E***A.sauteri*, brachypterous male **F***A.tsurugisana*, brachypterous male.

**Figure 5.**
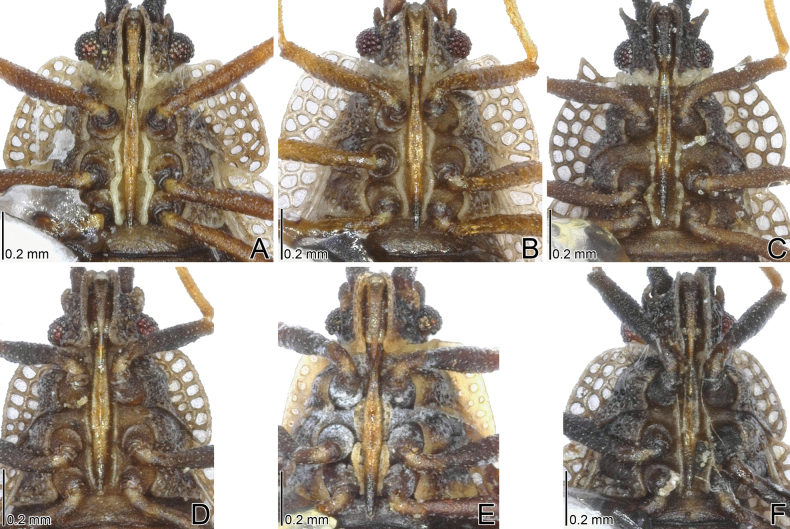
Rostra of six *Acalypta* species from Japan. **A***A.alutacea* sp. nov. **B***A.carinata***C***A.cooleyi***D***A.gracilis***E***A.hirashimai***F***A.marginata*.

Figs 2A, 4A, 5E, 8A, 9E, 11E, 15H

Japanese name: Hirashimamaru-gunbai

**Distribution.** Japan (Hokkaido) (Tomokuni 1983; Yamada and Tomokuni 2012; Yamada and Ishikawa 2016; Aukema 2025).

***Acalyptamarginata* (Wolff, 1804)**

Figs 2B, 4B, 5F, 8B, 9F, 11F, 15I

Japanese name: Yukigunimaru-gunbai

**Distribution.** Armenia(?), Austria, Azerbaijan, Belgium, Bosnia Herzegovina, Bulgaria, Byelorussia, Croatia, Czech Republic, Finland, France, Germany, Hungary, Italy, Japan (Hokkaido), Korea, Latvia, Luxembourg, Moldavia, Mongolia, Netherlands, Norway, Poland, Portugal, Romania, Russia, Serbia, Slovakia, Slovenia, Spain, Sweden, Switzerland, Turkey, Ukraine (Péricart and Golub 1996; Aukema et al. 2013; Cho et al. 2020; Souma 2021; Aukema 2025).

***Acalyptamiyamotoi* Takeya, 1962**

**Figure 6.**
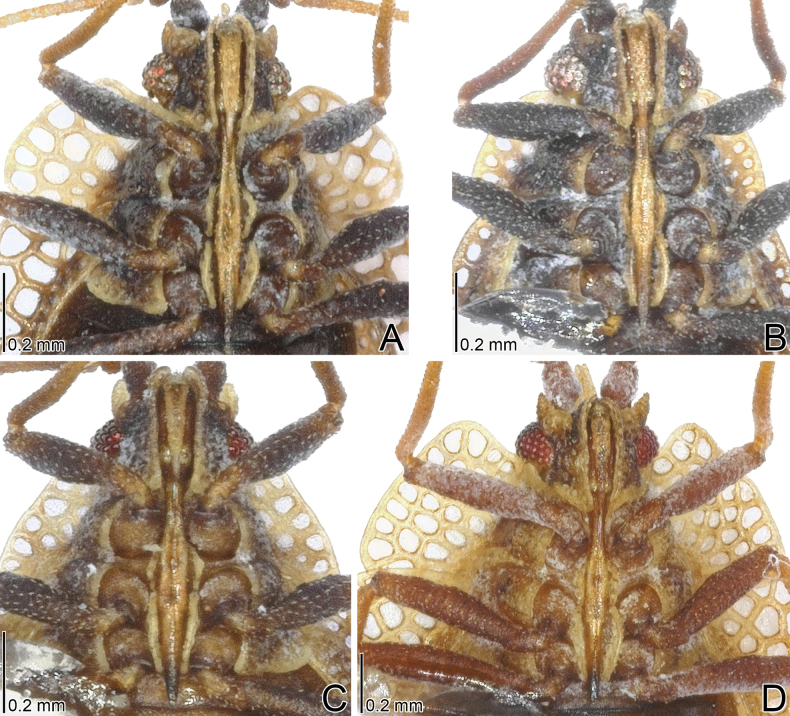
Rostra of four *Acalypta* species from Japan. **A***A.miyamotoi***B***A.pallidicoronata***C***A.sauteri***D***A.tsurugisana*.

Figs 2C, 4C, 6A, 8C, 10A, 12A, 16A

Japanese name: Miyamotomaru-gunbai

**Distribution.** Japan (Honshu, Shikoku, Kyushu) (Tomokuni 1983; Yamada and Tomokuni 2012; Yamada and Ishikawa 2016; Aukema 2025).

**Figure 7.**
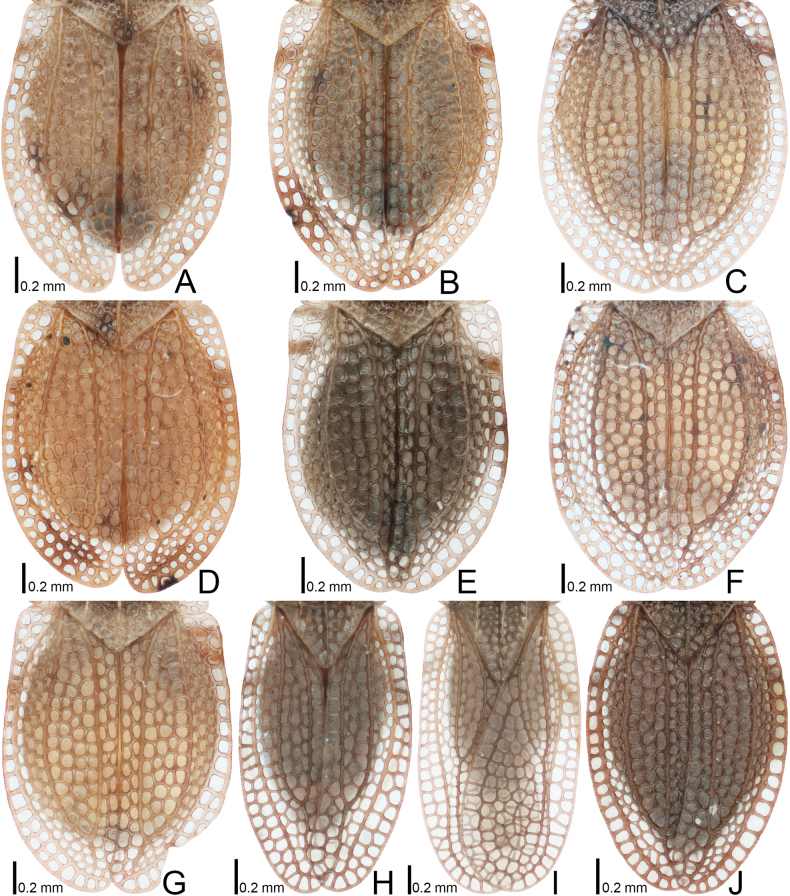
Hemelytra of four *Acalypta* species from Japan. **A, B***A.alutacea* sp. nov., brachypterous males **C, D***A.alutacea* sp. nov., brachypterous females **E***A.carinata*, brachypterous male **F, G***A.carinata*, brachypterous females **H***A.cooleyi*, brachypterous male **I***A.cooleyi*, macropterous male **J***A.gracilis*, brachypterous male.

**Figure 8.**
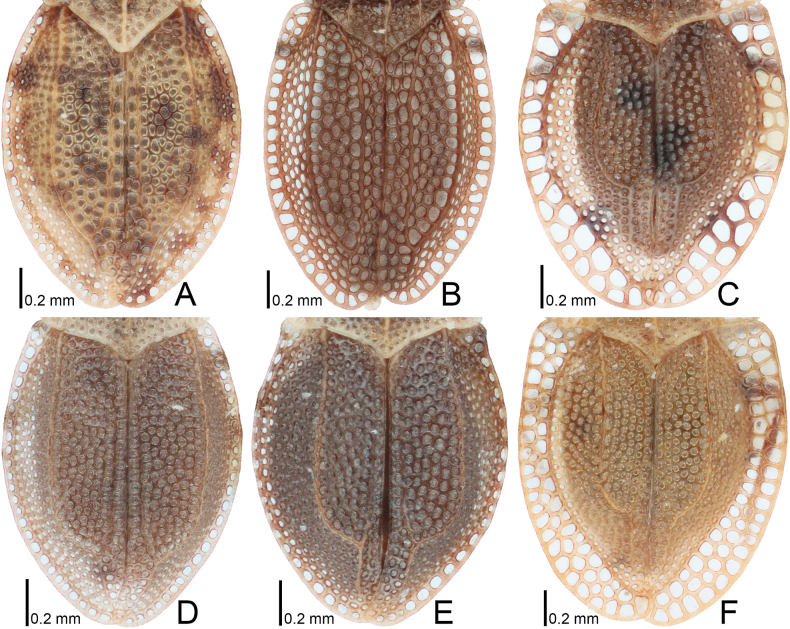
Hemelytra of six *Acalypta* species from Japan. **A***A.hirashimai*, brachypterous male **B***A.marginata*, brachypterous male **C***A.miyamotoi*, brachypterous male **D***A.pallidicoronata*, brachypterous male **E***A.sauteri*, brachypterous male **F***A.tsurugisana*, brachypterous male.

**Figure 9.**
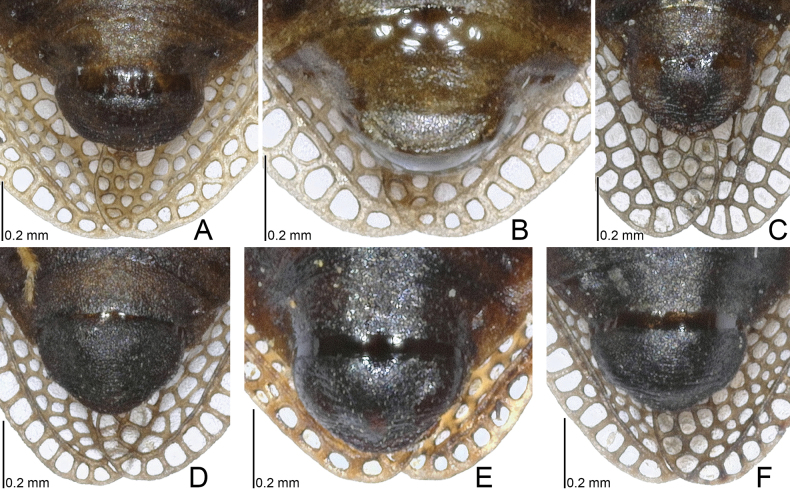
Male terminalia of six *Acalypta* species from Japan. **A***A.alutacea* sp. nov. **B***A.carinata***C***A.cooleyi***D***A.gracilis***E***A.hirashimai***F***A.marginata*.

**Figure 10.**
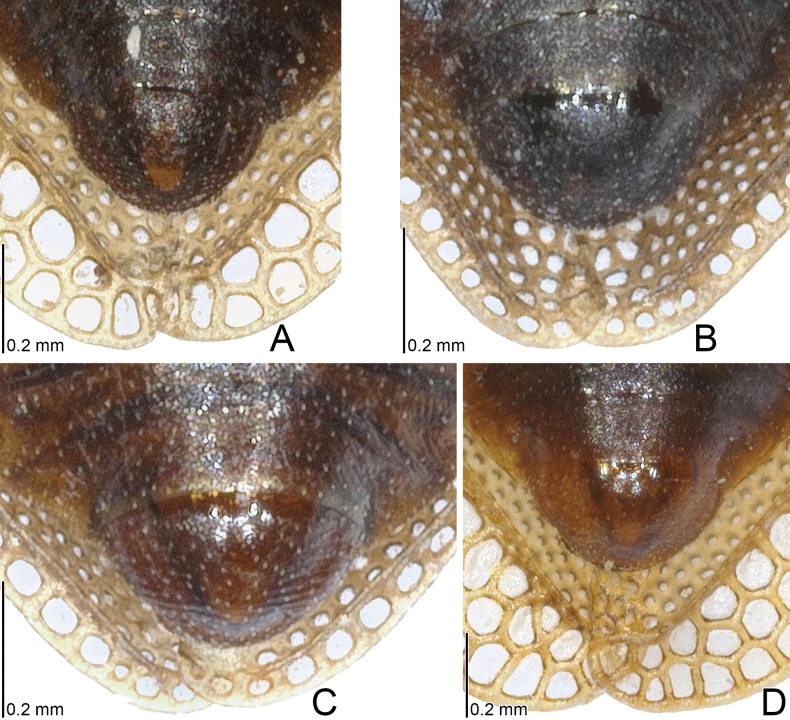
Male terminalia of four *Acalypta* species from Japan. **A***A.miyamotoi***B***A.pallidicoronata***C***A.sauteri***D***A.tsurugisana*.

**Figure 11.**
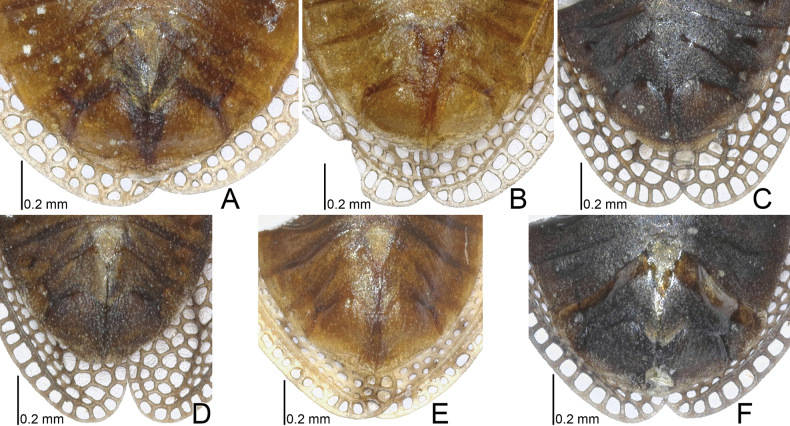
Female terminalia of six *Acalypta* species from Japan. **A***A.alutacea* sp. nov. **B***A.carinata***C***A.cooleyi***D***A.gracilis***E***A.hirashimai***F***A.marginata*.

**Figure 12.**
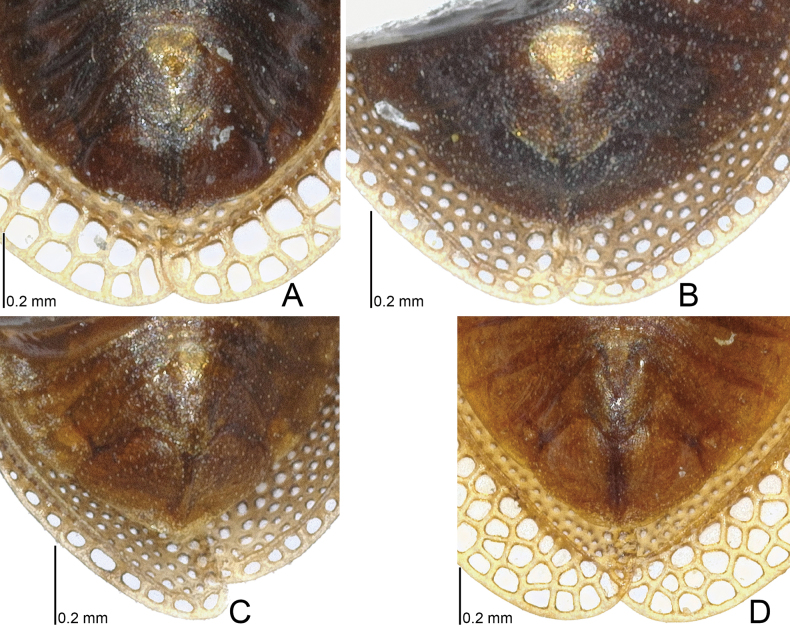
Female terminalia of four *Acalypta* species from Japan. **A***A.miyamotoi***B***A.pallidicoronata***C***A.sauteri***D***A.tsurugisana*.

***Acalyptapallidicoronata* Souma, 2019**

Figs 2D, 4D, 6B, 8D, 10B, 12B, 16B

Japanese name: Nagasakimaru-gunbai

**Distribution.** Japan (Hirado Island, Tsushima Island) (Souma 2019, 2023a; Aukema 2025).

***Acalyptasauteri* Drake, 1942**

Figs 2E, 4E, 6C, 8E, 10C, 12C, 16C, D

Japanese name: Maru-gunbai

**Distribution.** Japan (Hokkaido, Honshu, Awa Island, Sado Island, Awaji Island, Oki Islands (Dogo Island), Ikuchi Island, Shikoku, Shodo Island, Omi Island, Kyushu, Amakusa Islands (Shimoshima Island)) (Tomokuni 1983, 1985; Nozaki et al. 2016; Yamada and Tomokuni 2012; Yamada and Ishikawa 2016; Aukema 2025).

**Figure 13.**
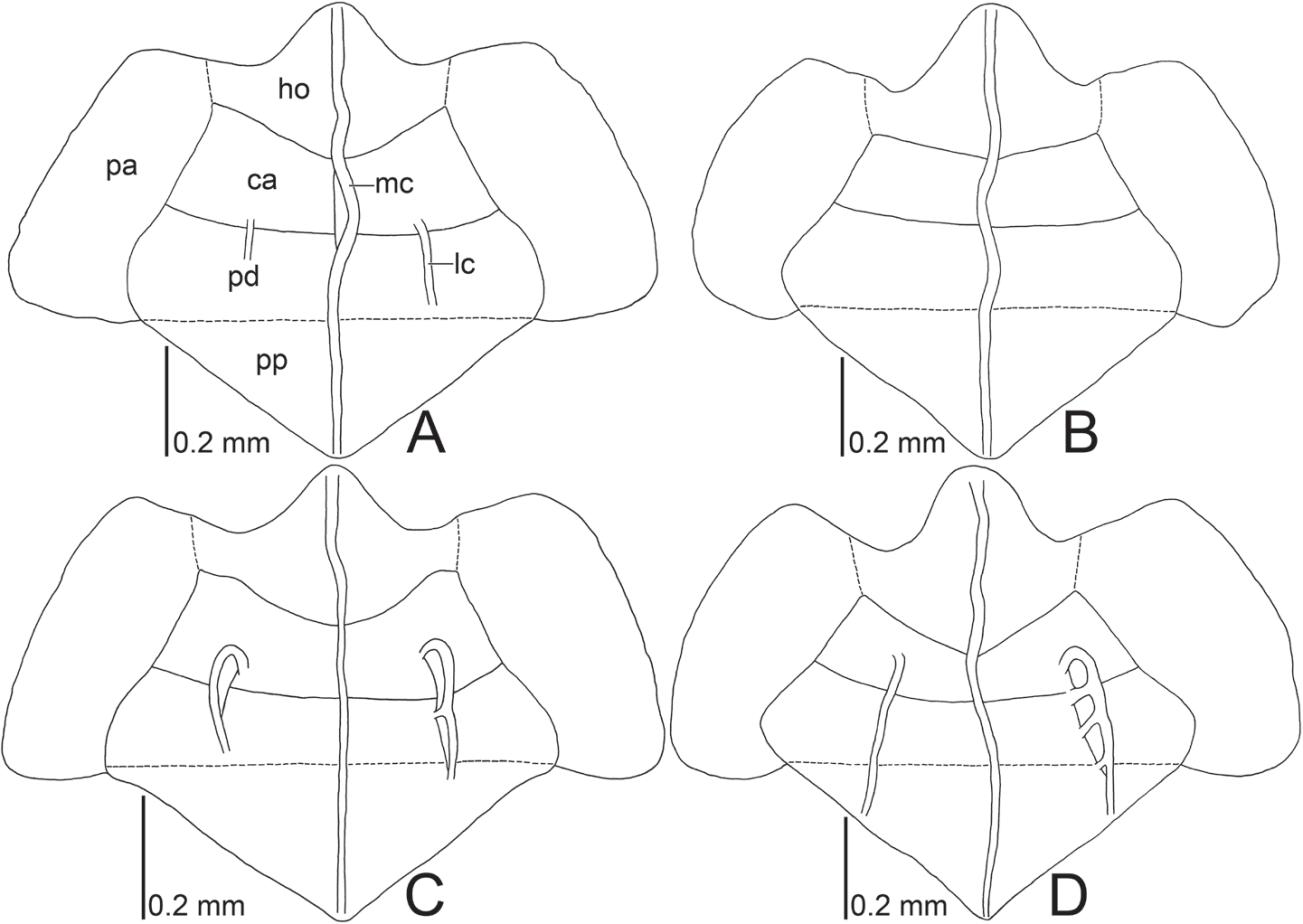
Line drawings of pronota of two *Acalypta* species from Japan, dorsal view. **A, B***A.alutacea* sp. nov. **C, D***A.carinata*. Abbreviations: ca, calli; ho, hood; lc, lateral carina; mc, median carina; pa, paranotum; pd, pronotal disc; pp, posterior process.

**Figure 14.**
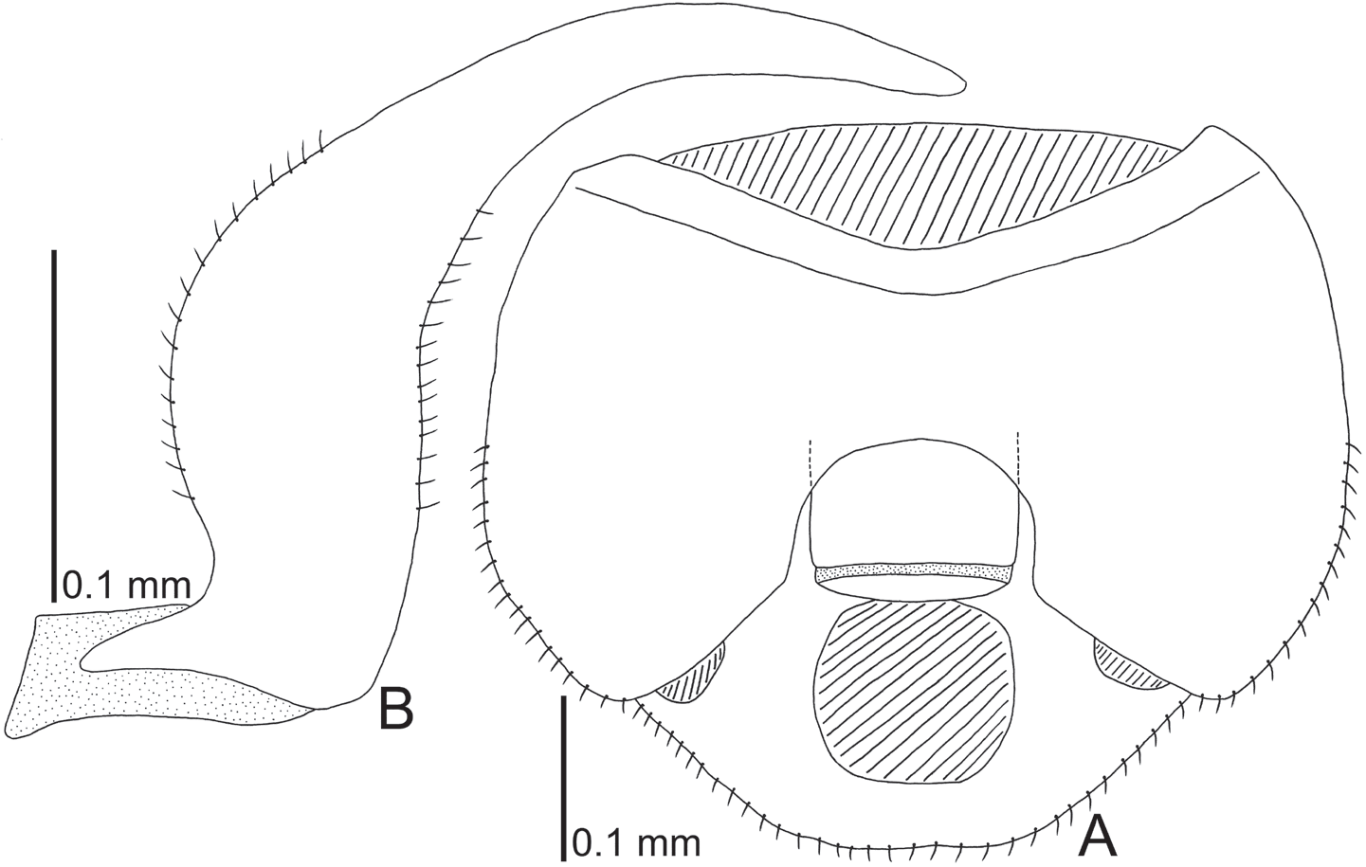
Line drawings of male genitalia of *A.alutacea* sp. nov., dorsal view. **A** pygophore **B** paramere.

**Figure 15.**
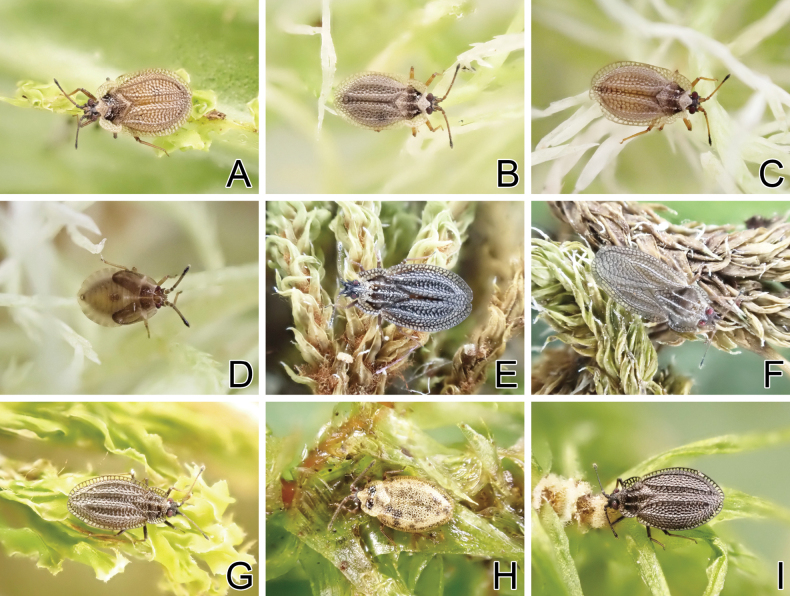
Living individuals of six *Acalypta* species from Japan. **A***A.alutacea* sp. nov., brachypterous morph **B, C***A.carinata*, brachypterous morphs **D***A.carinata*, fifth instar nymph **E***A.cooleyi*, brachypterous morph **F***A.cooleyi*, macropterous morph **G***A.gracilis*, brachypterous morph **H***A.hirashimai*, brachypterous morph **I***A.marginata*, brachypterous morph.

**Figure 16.**
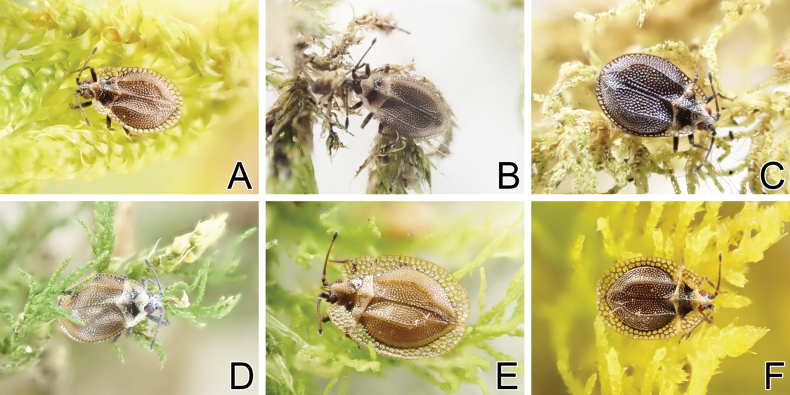
Living individuals of four *Acalypta* species from Japan. **A***A.miyamotoi*, brachypterous morph **B***A.pallidicoronata*, brachypterous morph **C, D***A.sauteri*, brachypterous morphs **E, F***A.tsurugisana*, brachypterous morphs.

**Figure 17.**
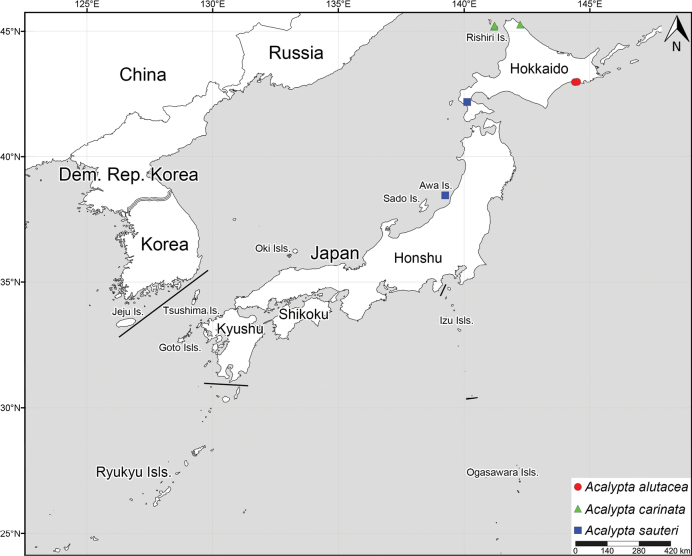
Collection sites of three *Acalypta* species from Japan used in present study.

***Acalyptatsurugisana* Tomokuni, 1972**

Figs 2F, 4F, 6D, 8F, 10D, 12D, 16E, F

Japanese name: Tsurugimaru-gunbai

**Distribution.** Japan (Honshu, Shikoku, Kyushu) (Tomokuni 1983; Yamada and Tomokuni 2012; Yamada and Ishikawa 2016; Souma 2023b; Aukema 2025).

### ﻿Key to the species of *Acalypta* occurring in Japan

Modified after the key provided by Souma (2021, 2023a).

**Table:** 

1	Pronotal disc as long as or longer than calli (Figs 1A–G, 2B, 3A–J, 4B); punctures on pronotal disc smaller than areolae of posterior process; Cu (cubitus) vein of hemelytron distinct throughout its length (Figs 7A–J, 8B)	**2**
–	Pronotal disc shorter than calli (Figs 2A, C–F, 4A, C–F); punctures on pronotal disc as large as areolae of posterior process; Cu vein of hemelytron indistinct in basal part and distinct in remaining parts (Fig. 8A, C–F)	**6**
2	Antenniferous tubercle acute, straight (Figs 1E, F, 3H, J); rostrum reaching posterior part of metasternum (Fig. 5C); anterolateral angle of paranotum angular; pronotal disc longer than calli in brachypterous morph; pygophore hexagonal, flat (Fig. 9C)	***A.cooleyi* Drake, 1917**
–	Antenniferous tubercle obtuse, curved inward (Figs 1A–D, G, 2B, 3A–G, J, 4B); rostrum reaching anterior part of abdominal sternite II (Fig. 5A, B, D, F); anterolateral angle of paranotum rounded; pronotal disc as long as calli in brachypterous morph; pygophore roundly inflated (Fig. 9A, B, D, F)	**3**
3	Paranotum as wide as hood (Fig. 3A–G); abdomen pale brown in female (Fig. 11A, B)	**4**
–	Paranotum narrower than hood (Figs 3J, 4B); abdomen dark brown in female (Fig. 11D, F)	**5**
4	Lateral carina of pronotum absent or reduced, shorter than hood, without or with a single minute areola; anterolateral angle of paranotum not or weakly protruding anteriorly, not reaching mid-level of compound eye (Figs 1A, B, 3A–D, 13A, B)	***A.alutacea* sp. nov.**
–	Lateral carina of pronotum developed, as long as hood, with a single row of areolae; anterolateral angle of paranotum strongly protruding anteriorly, reaching mid-level of compound eye (Figs 1C, D, 3E–G, 13C, D)	***A.carinata* (Panzer, 1806)**
5	Basal part of antennal segment III thickened (Fig. 1G)	***A.gracilis* (Fieber, 1844)**
–	Basal part of antennal segment III not thickened (Fig. 2B)	***A.marginata* (Wolff, 1804)**
6	Pronotum more than 3/4 times as long as maximum width across paranota (Figs 2A, D, 4A, D); discoidal area of hemelytron considerably expanded beyond apical fourth of hemelytron, distinctly wider than subcostal area (Fig. 8A, D)	**7**
–	Pronotum less than 3/4 times as long as maximum width across paranota (Figs 2C, E, F, 4C, E, F); discoidal area of hemelytron not expanded beyond apical fourth of hemelytron, not wider than subcostal area (Fig. 8C, E, F)	**8**
7	Hemelytron irregularly scattered with dark spots (Fig. 8A); posterolateral angle of paranotum protruding posteriorly (Figs 2A, 4A); posterior process of pronotum strongly protruding posteriorly, as long as hood	***A.hirashimai* Takeya, 1962**
–	Hemelytron without dark spots (Fig. 8D); posterolateral angle of paranotum not protruding posteriorly (Figs 2D, 4D); posterior process of pronotum weakly protruding posteriorly, shorter than hood	***A.pallidicoronata* Souma, 2019**
8	Paranotum narrowed posteriorly (Figs 2E, 4E); anterolateral angle of paranotum weakly protruding anteriorly, not reaching mid-level of compound eye; posterolateral angle of paranotum protruding posteriorly; costal area of hemelytron with 2 rows of areolae in basal part and a single row (rarely 2 rows) in apical part (Fig. 8E)	***A.sauteri* Drake, 1942**
–	Paranotum not narrowed posteriorly (Figs 2C, F, 4C, F); anterolateral angle of paranotum strongly protruding anteriorly, reaching mid-level of compound eye; posterolateral angle of paranotum not protruding posteriorly; costal area of hemelytron with 3–4 rows of areolae in basal part and 2–3 rows (rarely a single row) in apical part (Fig. 8C, F)	**9**
9	Paranotum with 3 rows of areolae throughout its length (Figs 2C, 4C); costal area of hemelytron with 3 rows of areolae in basal part, a single row in middle part, and 2 rows (rarely a single row) in apical part (Fig. 8C); discoidal area as wide as subcostal area at widest part	***A.miyamotoi* Takeya, 1962**
–	Paranotum with 4–5 rows of areolae throughout its length (Figs 2F, 4F); costal area of hemelytron with 4 rows of areolae in basal part, 2 rows in middle part, and 3 rows in apical part (Fig. 8F); discoidal area narrower than subcostal area at widest part	***A.tsurugisana* Tomokuni, 1972**

## Supplementary Material

XML Treatment for Acalypta

XML Treatment for Acalypta alutacea

XML Treatment for Acalypta carinata

XML Treatment for Acalypta sauteri

## ﻿Additional information

### Author ORCIDs

Jun Souma https://orcid.org/0000-0002-2238-5015

### Data availability

All of the data that support the findings of this study are available in the main text.
